# Shared genetic architecture between the two neurodegenerative diseases: Alzheimer’s disease and glaucoma

**DOI:** 10.3389/fnagi.2022.880576

**Published:** 2022-09-01

**Authors:** Chunwen Zheng, Shunming Liu, Xiayin Zhang, Yunyan Hu, Xianwen Shang, Zhuoting Zhu, Yu Huang, Guanrong Wu, Yu Xiao, Zijing Du, Yingying Liang, Daiyu Chen, Siwen Zang, Yijun Hu, Mingguang He, Xueli Zhang, Honghua Yu

**Affiliations:** ^1^Shantou University Medical College, Shantou, China; ^2^Guangdong Eye Institute, Department of Ophthalmology, Guangdong Provincial People’s Hospital, Guangdong Academy of Medical Sciences, Guangzhou, China; ^3^State Key Laboratory of Ophthalmology, Zhongshan Ophthalmic Center, Sun Yat-sen University, Guangzhou, China; ^4^Centre for Eye Research Australia, Royal Victorian Eye and Ear Hospital, East Melbourne, VIC, Australia; ^5^Medical Research Center, Guangdong Provincial People’s Hospital, Guangdong Academy of Medical Sciences, Guangzhou, China

**Keywords:** Alzheimer’s disease, glaucoma, shared genetic architecture, neurodegenerative diseases, genetic pleiotropy

## Abstract

**Background:**

Considered as the representatives of neurodegenerative diseases, Alzheimer’s disease (AD) and glaucoma are complex progressive neuropathies affected by both genetic and environmental risk factors and cause irreversible damages. Current research indicates that there are common features between AD and glaucoma in terms of epidemiology and pathophysiology. However, the understandings and explanations of their comorbidity and potential genetic overlaps are still limited and insufficient.

**Method:**

Genetic pleiotropy analysis was performed using large genome-wide association studies summary statistics of AD and glaucoma, with an independent cohort of glaucoma for replication. Conditional and conjunctional false discovery rate methods were applied to identify the shared loci. Biological function and network analysis, as well as the expression level analysis were performed to investigate the significance of the shared genes.

**Results:**

A significant positive genetic correlation between AD and glaucoma was identified, indicating that there were significant polygenetic overlaps. Forty-nine shared loci were identified and mapped to 11 shared protein-coding genes. Functional genomic analyses of the shared genes indicate their modulation of critical physiological processes in human cells, including those occurring in the mitochondria, nucleus, and cellular membranes. Most of the shared genes indicated a potential modulation of metabolic processes in human cells and tissues. Furthermore, human protein–protein interaction network analyses revealed that some of the shared genes, especially *MTCH2*, *NDUFS3*, and *PTPMT1*, as well as *SPI1* and *MYBPC3*, may function concordantly. The modulation of their expressions may be related to metabolic dysfunction and pathogenic processes.

**Conclusion:**

Our study identified a shared genetic architecture between AD and glaucoma, which may explain their shared features in epidemiology and pathophysiology. The potential involvement of these shared genes in molecular and cellular processes reflects the “inter-organ crosstalk” between AD and glaucoma. These results may serve as a genetic basis for the development of innovative and effective therapeutics for AD, glaucoma, and other neurodegenerative diseases.

## Introduction

With an increasingly aging population, neurodegenerative diseases have inevitably brought great challenges to society and people on a global level. This demographic shift and the development of neurodegenerative diseases are not only followed by a deterioration of cognitive function and the impediment of independence for individuals but also by an overall exacerbation of the health burden on the society (Mancino et al., 2018; Gupta et al., 2021; Nicholas et al., 2021). As the typical representatives of neurodegenerative diseases, Alzheimer’s disease (AD) is considered the most frequent type, accounting for 60–70% of dementia cases (Hansson, 2021), while glaucoma is the most common cause of irreversible blindness and the most common type of optic neuropathy (Quigley and Broman, 2006; Chang and Goldberg, 2012). Because of the severity and universality of these diseases, both serve as critical points for further study in other neurodegenerative diseases.

Recent studies have highlighted that there are connections between AD and glaucoma, suggesting that AD and glaucoma could be considered the age-related neurodegenerative diseases that may coexist in the aging population (Mirzaei et al., 2017; Giorgio et al., 2018; Mancino et al., 2018). This phenomenon could be deduced because these two neurodegenerative diseases do share some similarities at both epidemiologic and pathophysiologic levels, with potential inter-organ crosstalk between brain and eye. From an epidemiologic perspective, there is evidence that AD patients would have a significantly higher rate of glaucoma occurrence (Chandra et al., 1986; Helmer et al., 2013; Mancino et al., 2018). Chandra et al. first reported an increased prevalence of glaucoma among senile and pre-senile dementia patients (Chandra et al., 1986). This was supported by other studies, which found a nearly sixfold higher prevalence of glaucoma in AD patients than in the control group (Bayer et al., 2002a,b). Similarly, a higher frequency of glaucoma has been reported in dementia patients in other studies. Tamura et al. (2006) found that Japanese patients with AD had a higher prevalence of open-angle glaucoma (Elyashiv et al., 2014). Furthermore, a large epidemiological study performed in the European population also found indications of a positive clinical correlation between the two diseases (Jefferis et al., 2013).

While investigating the relationship between these two diseases from a pathophysiological perspective, scientists found that AD and glaucoma both affect the elderly, causing chronic and progressive irreversible cell death, wherein the process of neurodegeneration and aging is accompanied by metabolic dysfunction. Common features, such as amyloid-β (Aβ) protein accumulation or aggregation, tau protein aggregation, and hyperphosphorylation, are now known to be shared between AD and glaucoma (Criscuolo et al., 2017). As a classic mechanism of AD development, the aggregation and accumulation of Aβ and tau proteins is then followed by a series of metabolic dysfunction processes (Origlia et al., 2010). Coincidentally, in glaucoma, the synaptic circuitry and retrograde trafficking of neurotrophic factors are also impaired by increased retinal levels of soluble Aβ protein (Poon et al., 2011; Della Santina et al., 2013; Gupta et al., 2014). Meanwhile, one of the known causes of glaucoma, namely high intraocular pressure, is also thought to induce Aβ protein expression, leading to an early reversible phase of retinal degeneration (McKinnon et al., 2002; Guo et al., 2007). Tau protein is also a neuronal toxicant that aggregates to result in AD (Engelborghs et al., 2008; Sahara et al., 2008; Fá et al., 2016). Similarly, phosphorylated tau protein was also found in aged retina and glaucoma (Lieven et al., 2007; Gasparini et al., 2011; Bull et al., 2012). In addition to these AD-like pathological changes in glaucoma, there are some glaucoma-like pathological changes in AD. As an optic neuropathy, glaucoma is characterized by retinal ganglion cell death and a thinning of the nerve fiber layer (Mancino et al., 2018). Interestingly, these changes have also been observed in AD patients. In addition to the affected neurons in the brain, widespread axonal degeneration has been observed in the optic nerves, as well as loss of retinal cells, especially ganglion cells in AD patients (Hinton et al., 1986; Quigley et al., 1989; Sadun and Bassi, 1990; Weinreb and Khaw, 2004). These findings illustrate the shared mechanisms between AD and glaucoma on pathophysiologic levels and serve as the basis for clinical therapies.

Nevertheless, similarities in epidemiology and pathophysiology remain unexplored in terms of their explanations and applications. Our primary challenge in dealing with these two representative neurodegenerative diseases is a scarce understanding of AD and glaucoma. Cumulative evidence indicates that the neuropathological process of AD and glaucoma is initiated several years before any clinical manifestations or diagnosis in patients (Ntsapi et al., 2018; Hansson, 2021). However, therapies are more likely to be effective at early pre-symptomatic or prodromal stages than when clinical manifestation and irreversible neurodegeneration occur (Hansson, 2021). Thus, determining the neurodegenerative mechanisms of AD and glaucoma is crucial for the exploration and development of new therapeutics.

With the growing knowledge of AD and glaucoma, there are increasing genome-wide association studies (GWASs) and related studies that have revealed several genetic variants affecting the two diseases (Tanzi and Bertram, 2001; Miller et al., 2004; Fan and Wiggs, 2010; Fingert, 2011; Bettens et al., 2013; Gharahkhani et al., 2021). More than 20 AD risk genes have been identified; however, the complete mechanisms through which these genes modulate disease remain unclear (Tanzi, 2012; Iadecola, 2013; Pruzin et al., 2017; Rojas-Gutierrez et al., 2017; Apostolova et al., 2018), with a similar condition was also being observed in glaucoma research (Janssen et al., 2013; Wiggs and Pasquale, 2017). A recent review mentioned that a large number of genes, such as *CAV1*, *CDKN2B*, and *GAS7*, were found to be involved in the development of glaucoma in recent GWASs (Janssen et al., 2013). *ANGPT1* and *SVEP1* have also been investigated and are considered promising candidate molecules for novel glaucoma therapies (Thomson et al., 2021). *GLIS1* was shown to be associated with glaucoma by regulating and maintaining trabecular meshwork function (Nair et al., 2021). Although there is an overlap in the epidemiology and pathophysiology between AD and glaucoma, whether the shared features of the two diseases could be explained genetically, that is, whether they are genetically related, remains unknown. At present, the potential shared genetic architecture between AD and glaucoma remains inconclusive, and further investigation is warranted.

This study aimed to elucidate the shared genetic architecture and the underlying molecular mechanisms of AD and glaucoma. By investigating the overlapping genetic loci and candidate genes, we aimed to identify the biological relationship between AD and glaucoma, and finally provide an explanation for the shared epidemiologic and pathophysiologic features of these two representative neurodegenerative diseases.

## Material and methods

### Genome-wide association study datasets for the discovery sample and replication sample

Genome-wide association study summary statistics of AD and glaucoma were obtained from publicly accessible websites.^1^ The factors for selection include the publication journal, names of the reported traits, numbers of samples used, race of the population, integrity of the datasets, and quality of the uploaded data. The summary statistics of AD (GCST002245) were obtained from a meta-analysis published by Lambert et al. (2013), who identified 11 new susceptibility loci of AD from 74,046 European ancestry, which were based on 7,055,881 single nucleotide polymorphisms (SNPs) and consisted of 17,008 AD cases and 37,154 controls. The summary statistics for the discovery sample (GCST90018852) of glaucoma were obtained from GWAS containing 25,844,939 SNPs with 10,411 European ancestry cases, 474,568 European ancestry controls, 8,448 East Asian ancestry cases, and 168,903 East Asian ancestry controls (Sakaue et al., 2021). To confirm the findings in the discovery sample, a replication sample of glaucoma was assessed. The second summary statistics as a replication sample (GCST009722) of glaucoma were obtained from a multi-trait analysis with a discovery sample of 7,947 cases and 119,318 controls, and a replication sample of 6,924 cases and 40,230 controls, which contained 8,002,429 SNPs (Supplementary Table 1) (Craig et al., 2020). Detailed study designs, including sample collection, quality control, and method imputation, were reported in each publication. The threshold of significant SNP was set as E−05; hence, SNPs with *P* < 1.0 × 10^–5^ were considered statistically significant. A workflow of the analysis process of this study is shown in Supplementary Figure 1.

### Quality control and imputation

For quality control, the details of the study-specific design, which include sample collection, quality control procedures, and SNPs inclusion or exclusion criteria, have been described in the corresponding publication of each GWASs (Lambert et al., 2013; Craig et al., 2020; Sakaue et al., 2021). Overall, SNPs with call rates <95% and with a minor allele frequency (MAF) of <1% were also excluded. SNPs with *P*-value that had significant deviations from the Hardy–Weinberg equilibrium (<1.0 × 10^–6^) were excluded. Software used for imputation were Minimac3, SNP2HLA, etc.

### Pleiotropy analysis

#### Conditional quantile–quantile plots

Quantile–quantile (Q-Q) plots were applied to show the SNPs association between a primary phenotype (e.g., AD) and a conditional phenotype (e.g., glaucoma). The pleiotropy analysis strategy of conditional Q-Q plots has been described in detail in previous reports (Liu et al., 2013; Andreassen et al., 2015; Desikan et al., 2015). Pleiotropic enrichment of AD conditional on glaucoma exists if the proportion of significant AD SNPs increases as a function of increased significant glaucoma SNPs, and vice versa. In our study, pleiotropic enrichment analysis was performed in both directions, with AD as the primary phenotype on glaucoma as the conditional phenotype, followed by the interchange of the primary and conditional phenotypes. Fold enrichment plots were graphically depicted to show the stratified SNPs relationship between AD and glaucoma using empirical quantiles of nominal −log_10_(p) values. The nominal −log_10_(p) values for the primary phenotype were plotted on the *X*-axis, while the fold enrichment, which shows the function between the primary and conditional phenotypes, was plotted on the *Y*-axis. Different cut-offs of *P*-values were delineated by individual lines with different colors. The cross-trait enrichment was represented as a successive leftward shift from the null line. Analyses were performed using the R software (version 4.1.1).

#### Conditional and conjunction false discovery rates

Conditional and conjunctional false discovery rates (FDRs) (cond and conjFDR, respectively) were applied to investigate the specific pleiotropic SNPs that were significant in both AD and glaucoma. The FDR is a statistical approach for the correction in multiple hypothesis testing, which was used in pleiotropy analysis to reflect the possibility of non-pleiotropy for an SNP (Smeland et al., 2020).

$$FDR\left(p_{i}\right)=Pr\left(H_{0}^{\left(i\right)}|P_{i}\leq p_{i}\right)$$

where *P*_*i*_ is the random variable of the *P*-value for trait *i* among all SNPs, and *p*_*i*_ is the instance of *P*_*i*_ to a specific SNP. *H*_0_^(^*^i^*^)^ represents the null hypothesis that a specific SNP is not associated with trait *i*.

Based on the Bayesian conditional false discovery rate-based (condFDR) method, the condFDR method was applied to increase the power of detecting pleiotropy associated with the primary phenotype (e.g., AD) by leveraging that associated with the second phenotype (e.g., glaucoma) (Andreassen et al., 2013, 2015; Liley and Wallace, 2015). CondFDR is an extension of the FDR calculations and uses the associations between genetic variants and the secondary phenotype to re-rank the *P*-values for the primary phenotype. We performed condFDR analysis to discover genetic variants associated with AD conditional on glaucoma. If the primary phenotype (e.g., AD) and the second phenotype (e.g., glaucoma) are genetically related, the condFDR will reorder the SNPs with a different ranking from the order of the primary phenotype alone (Desikan et al., 2015). The condFDR of the principal phenotype (e.g., AD) conditioned on glaucoma (AD| glaucoma) and the reversing condFDR (glaucoma| AD) were calculated as follows:

$$cFDR\left(p_{i}|p_{j}\right)=Pr\left(H_{0}^{\left(i\right)}|P_{i}\leq p_{i},P_{j}\leq p_{j}\right)$$

where *p*_*i*_ is the association of a specific SNP with the principal disease, *p*_*j*_ is with the conditional disease.

In order to find the pleiotropic SNPs that are jointly significant in the two phenotypes, conjFDR, an extension of condFDR, was calculated (Witoelar et al., 2017). ConjFDR is defined as the greater of the two condFDR values and it serve a conservative estimation of the FDR for a pleiotropic SNP associated with both phenotypes. In our study, we performed conjFDR analysis to identify the shared SNPs between AD and glaucoma based on the previously calculated condFDR. The threshold conjFDR < 0.01 was set, and *P*-values were corrected by genomic inflation control procedure as described in other studies (Andreassen et al., 2013; Johnston et al., 2019; Cheng et al., 2021). For SNPs with multiple ccFDR values, the average values were employed. R (version 4.1.1) was used to calculate the cFDR algorithm using the KehaoWu/GWAScFDR package.^2^

$$c\,c\,F\,D\,R_{i\&j}=m\,a\,x\,\left(c\,F\,D\,R_{i|j},c\,F\,D\,R_{j|i}\right)$$

ccFDR (conjFDR), where ccFDR*_*i*_*_&_*_*j*_* is the maximum value of cFDR (*i*| *j*) and cFDR(*j*| *i*).

### Functional interpretation and analysis of shared loci and genes

The individual pleiotropic SNPs were mapped to their corresponding genes using GeneCards (The Human Gene Database^3^). The gene context of each significant SNP was examined in the National Center for Biotechnology Information (NCBI) database.^3^

The STRING database^4^ (version 11.5) (Szklarczyk et al., 2019) was used to map the shared genes between AD and glaucoma on the human protein--protein interaction (PPI) network to identify the potential pathways shared by both AD and glaucoma development. Metascape^5^ was also used for finding potential enrichment pathways. Gene-set enrichment, including biological process, molecular function, cellular compartment, and subcellular localization, was analyzed using Gene Ontology (GO), Kyoto Encyclopedia of Genes and Genomes (KEGG) Pathways, Database for Annotation, Visualization and Integrated Discovery (DAVID), and COMPARTMENTS. The adjusted FDR *P*-value was < 0.05 and the earlier pathway selection threshold was set at 0.05. Gene expression analysis and clustering of shared pleiotropic genes were performed using the Genotype-Tissue Expression (GTEx) database.^6^ The shared gene expression in different human tissues and their single-cell expression level in various human cells and tissues were analyzed using a heatmap.

### Code availability

Publicly available software was applied for the analyses, and the corresponding programs used were listed and described above.

### Statistical analysis

Details of the statistical analysis were mentioned in each section above. cond/conjFDR was computed to identify the risk loci associated with AD and glaucoma. The threshold of significant SNP was set as E−05 and the threshold of conjFDR was set as <0.01. Conditional Q-Q plot was applied to assess the pleiotropic enrichment, and different cut-offs of *P*-values were delineated by individual lines in the figures. To confirm the findings in the AD and glaucoma discovery sample, we further assessed the *P*-values of the identified pleiotropic SNPs in the glaucoma replication sample. Analyses were mainly based on R (v4.1.1) and String database (v11.5).

## Results

### Genome-wide association study results for Alzheimer’s disease and glaucoma

Before assessing the polygenetic overlaps between AD and glaucoma, the individual GWAS results for each phenotype were compared for database quality control and overall genetic association statistics. After considering the sample size, sample recruitment, and publication data, GCST002245, GCST90018852, and GCST009722 were chosen as AD sample, glaucoma discovery sample, and glaucoma replication sample, respectively.

The summary statistics of AD (GCST002245) were from a two stages meta-analysis of 74,046 European ancestry individuals published by Lambert et al. (2013). The stage 1 was based on 7,055,881 SNPs, comprising 17,008 AD cases and 37,154 controls. The stage 2 was based on 11,632 SNPs and consisted of 8,572 AD cases, and 11,312 controls. The data used in this study were obtained from several consortia, and the corresponding information about the sex and age of the datasets was described in the study.

The summary statistics for the discovery sample (GCST90018852) of glaucoma included 25,844,939 SNPs from 10,411 European ancestry cases, and 474,568 European ancestry controls, 8,448 East Asian ancestry cases, and 168,903 East Asian ancestry controls (Sakaue et al., 2021). The data presented in this study were from two GWAS datasets with a mean age of participants 63.0 years old and 46.3% females, with a mean age of 56.8 years old and 53.8% females, respectively.

The summary statistics for the replication sample (GCST009722) of glaucoma contain 8,002,429 SNPs from a discovery sample of 7,947 cases and 119,318 controls, and a replication sample of 6,924 cases and 40,230 controls (Craig et al., 2020). Because the study was a GWAS from multiple datasets, detailed information on individual studies is provided in its supplementary note.

### Polygenic overlap between Alzheimer’s disease and glaucoma

Given the current epidemiological and pathophysiologic understandings of AD and glaucoma, these two neurodegenerative diseases share some similarities and have “inter-organ crosstalk.” As such, we focused on investigating and identifying the genetic overlaps between AD and glaucoma using GWAS summary statistics.

**FIGURE 1:**
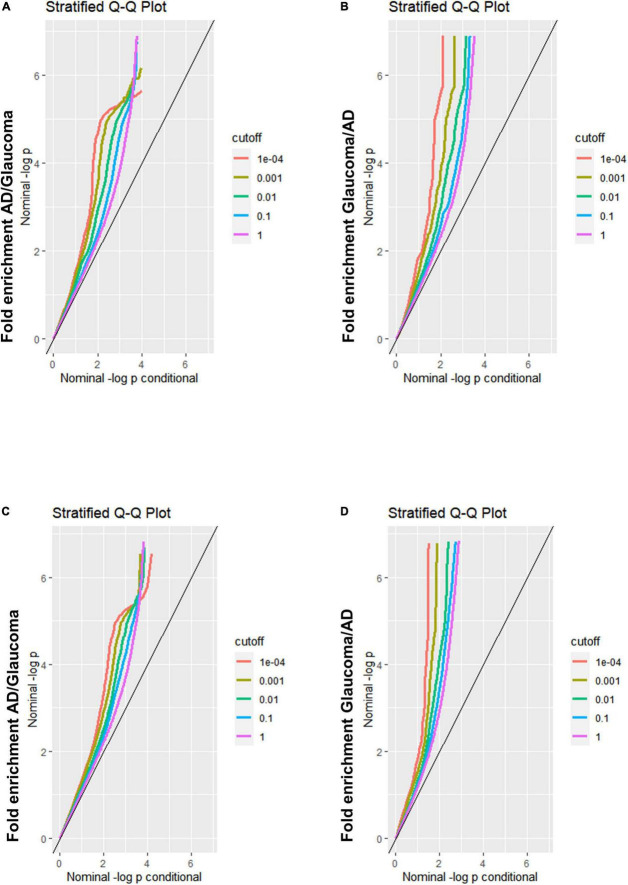
Fold-enrichment plots for genetic association analysis between AD and glaucoma discovery/replication samples. **(A)** Fold-enrichment plot of nominal –log_10_(p) for SNPs association with AD as a function of statistical significance for SNPs association with glaucoma discovery sample. The straight lines with sharp upward deviated tails showed the true associations between AD and glaucoma discovery sample. **(B)** Fold-enrichment plot of nominal –log_10_(p) for SNPs association with glaucoma discovery sample as a function of statistical significance for SNPs association with AD. The straight lines with sharp upward deviated tails showed the true associations between glaucoma discovery sample and AD. **(C)** Fold-enrichment plot of nominal –log_10_(p) for SNPs association with AD as a function of statistical significance for SNPs association with glaucoma replication sample. The straight lines with sharp upward deviated tails showed the true associations between AD and glaucoma replication sample. **(D)** Fold-enrichment plot of nominal –log_10_(p) for SNPs association with glaucoma replication sample as a function of statistical significance for SNPs association with AD. The straight lines with sharp upward deviated tails showed the true associations between glaucoma replication sample and AD.

A Q-Q plot was used to display the expected association between AD and glaucoma. If the Q-Q plot has straight lines with sharp upward deviated tails, it indicates that there are true associations between the two diseases. In the fold enrichment plots which were demonstrated as stratified conditional Q-Q plots, enrichment was observed in SNPs associated with AD across increasingly stringent levels of significance for SNPs associated with glaucoma [Q-Q(AD| Glaucoma)] (Figure 1A) between AD and glaucoma discovery sample. The reverse conditional association [Q-Q(Glaucoma| AD)] also showed a significant leftward shift at different cut-off lines (Figure 1B). These results indicated that there were significant polygenetic overlaps between AD and glaucoma. For validation of the results from the discovery sample, replication was performed. The fold enrichment plot again showed that there was significant enrichment for SNPs associated with AD across increasingly stringent levels of significance for those with glaucoma [Q-Q(AD| Glaucoma)] (Figure 1C) between AD and glaucoma replication sample. Similarly, the reverse conditional association [Q-Q(Glaucoma| AD)] demonstrated a significant enrichment between AD and glaucoma (Figure 1D). Therefore, both the discovery and replication results showed a significant polygenic overlap between AD and glaucoma.

The cond/conjFDR (ccFDR) methodology was performed to increase genetic discovery power and discover specific genetic variants, that is, shared loci, associated with AD conditional on glaucoma. At ccFDR < 0.01, 49 shared loci associated with AD and glaucoma phenotypes, including loci that were not significant (*P* < 5.0E−08) in the traditional AD GWAS (Table 1), were identified (Smeland et al., 2020). Detailed information, including the SNP name, genomic position, and closest gene, were shown in Table 1. These 49 identified shared loci were suggestively significant (*P* < 1.0E−05) for both phenotypes. These results indicated significant genetic associations between AD and glaucoma phenotypes.

**TABLE 1:** Shared pleiotropic loci between AD and glaucoma.

#	SNP	Genomic position	Closest gene	ccFDR
1	rs2856661	11:47374998	SPI1, MYBPC3	2.59E−05
2	rs11039199	11:47379212	SPI1	9.99E−06
3	rs7105851	11:47383275	SPI1	1.94E−05
4	rs11039203	11:47384597	SPI1	2.00E−05
5	rs67116452	11:47385194	SPI1	1.41E−05
6	rs10838698	11:47385923	SPI1	3.81E−05
7	rs10838699	11:47386073	SPI1	2.46E−05
8	rs935914	11:47387599	SPI1	1.45E−05
9	rs35996350	11:47389223	SPI1	2.25E−05
10	rs4752827	11:47389676	SPI1	3.40E−05
11	rs4752987	11:47390692	SPI1	1.16E−05
12	rs10769258	11:47391039	SPI1	1.29E−05
13	rs11039212	11:47393303	SPI1	2.12E−05
14	rs7940536	11:47395240	SPI1	2.83E−05
15	rs56030824	11:47397353	SPI1	1.17E−05
16	rs55677087	11:47397714	SPI1	1.38E−05
17	rs12146565	11:47398963	SPI1	2.26E−05
18	rs11601173	11:47401058	SPI1	1.30E−05
19	rs11607981	11:47401060	SPI1	1.37E−05
20	rs7111957	11:47409212	SPI1	1.57E−05
21	rs4752990	11:47410393	SPI1	1.29E−05
22	rs4752993	11:47410951	SPI1	1.25E−05
23	rs4752994	11:47411065	SPI1	5.90E−05
24	rs10769262	11:47411581	SPI1	3.61E−05
25	rs12803525	11:47413475	SPI1	1.02E−05
26	rs12802273	11:47418306	SPI1	1.83E−05
27	rs58965622	11:47418465	SPI1	9.86E−06
28	rs7924485	11:47419129	SPI1	1.38E−05
29	rs1317164	11:47419757	MTCH2	9.46E−06
30	rs4752832	11:47423553	MTCH2	1.21E−05
31	rs11039219	11:47424400	MTCH2	1.12E−05
32	rs755554	11:47432034	SLC39A13	1.38E−05
33	rs11600581	11:47448497	PSMC3	1.07E−05
34	rs2868459	11:47454972	MTCH2	1.32E−05
35	rs7106956	11:47458765	MTCH2	1.96E−05
36	rs35705029	11:47459963	RAPSN	9.83E−06
37	rs4752845	11:47539697	CELF1	9.17E−06
38	rs34958982	11:47547046	CELF1	1.73E−05
39	rs66749409	11:47568074	CELF1	1.94E−05
40	rs12798346	11:47583121	CELF1	2.08E−05
41	rs56400411	11:47586376	CELF1, PTPMT1	1.61E−05
42	rs7945473	11:47589707	PTPMT1	1.33E−05
43	rs2030166	11:47602729	NDUFS3	1.85E−05
44	rs11605348	11:47606483	FAM180B, NDUFS3	2.65E−05
45	rs4752856	11:47648042	MTCH2	1.53E−05
46	rs4752857	11:47655752	MTCH2	2.12E−05
47	rs11039327	11:47674084	MTCH2	8.99E−06
48	rs11604825	11:47725306	AGBL2	1.55E−05
49	rs11602395	11:47726977	AGBL2	8.76E−06

Forty-nine pleiotropic loci reach statistically significant ccFDR (<0.01) and are shared by AD and glaucoma phenotypes.

**FIGURE 2:**
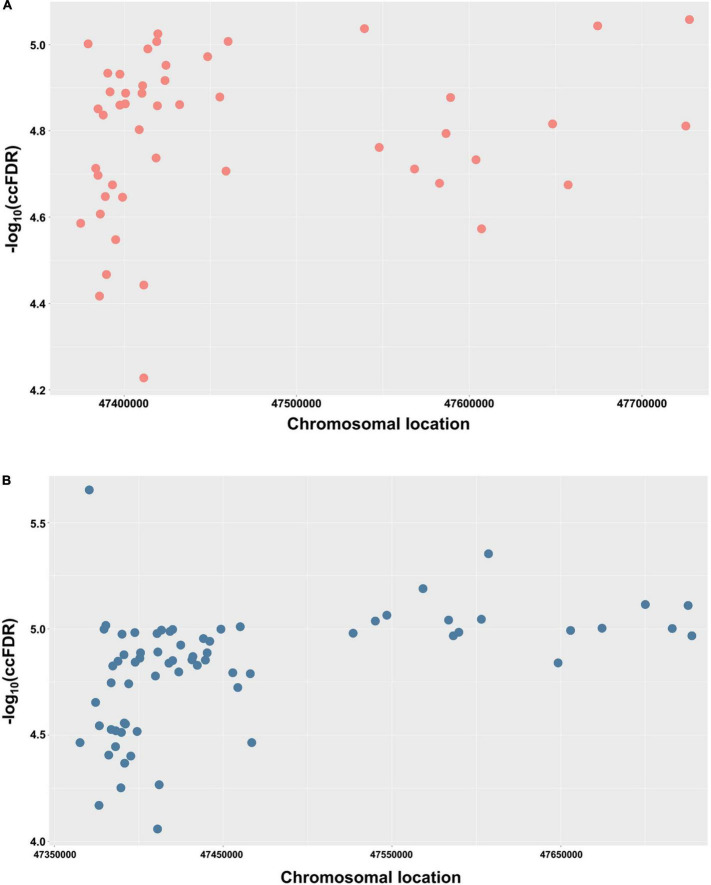
Conjunctional Manhattan plots depict the chromosomal location of each tested shared genetic loci between AD and glaucoma. **(A)** Conjunctional Manhattan plot of chromosomal locations and –log_10_(ccFDR) values for associated SNPs of AD and glaucoma discovery sample. **(B)** Conjunctional Manhattan plot of chromosomal locations and –log_10_(ccFDR) values for associated SNPs of AD and glaucoma replication sample. The *X*-axis is the chromosomal locations of significant shared loci. The *Y*-axis is the calculated –log_10_(ccFDR) of significant shared loci. Detailed information about chromosomal locations for all significant loci is shown in Table 1.

In order to demonstrate the chromosomal location of the significant shared SNPs, a Manhattan plot was used. For simplicity and clarity, we constructed a Manhattan plot using the chromosomal location of the significant shared SNPs along the *X*-axis and the negative log-10 of their ccFDR values on the *Y*-axis (Figure 2A). Similarly, to further confirm the results from the discovery sample, an independent replication sample was used for plotting (Figure 2B). A collection of significant SNPs on chromosome 11 were observed in the discovery and replication samples. There were 49 SNPs mapped to 11 proximal genes (*AGBL2*, *CELF1*, *FAM180B*, *MTCH2*, *MYBPC3*, *NDUFS3*, *PSMC3*, *PTPMT1*, *RAPSN*, *SLC39A13*, and *SPI1*), which were considered as the shared genes between AD and glaucoma (Table 2).

**TABLE 2:** Shared genes between AD and glaucoma.

Gene name abbreviation	Full gene name	Function of gene
AGBL2	AGBL carboxypeptidase 2	Catalyzes the deglutamylation of polyglutamate side chains generated by post-translational polyglutamylation in proteins
CELF1	CUGBP Elav-like family member 1	Pre-mRNA alternative splicing, mRNA translation and stability
FAM180B	Family with sequence similarity 180 member B	Enables protein binding
MTCH2	Mitochondrial carrier 2	Induces mitochondrial depolarization
MYBPC3	Myosin binding protein C3	Modifies the activity of actin-activated myosin ATPase.
NDUFS3	NADH:ubiquinone oxidoreductase core subunit S3	Core subunit of the mitochondrial membrane respiratory chain NADH dehydrogenase (Complex I)
PSMC3	Proteasome 26S subunit, ATPase 3	Maintenance of protein homeostasis by removing misfolded or damaged proteins
PTPMT1	Protein tyrosine phosphatase mitochondrial 1	Prevent intrinsic apoptosis, probably by regulating mitochondrial membrane integrity
RAPSN	Receptor associated protein of the synapse	Postsynaptic protein required for clustering of nicotinic acetylcholine receptors (nAChRs) at the neuromuscular junction
SLC39A13	Solute carrier family 39 member 13	Transmembrane protein functions as a zinc transporter
SPI1	Spi-1 proto-oncogene	Transcriptional activator that may be specifically involved in the differentiation or activation of macrophages or B-cells

Eleven genes mapped by 49 shared pleiotropic loci are considered as the shared genes between AD and glaucoma.

### Assessment of biological function and network analysis of the pleiotropic genes shared by Alzheimer’s disease and glaucoma

After identifying the significant shared loci between AD and glaucoma using discovery and replication samples, we further conducted bioinformatics analyses to investigate the biological mechanisms through which these shared loci or genes modulate the pathogenesis of the two diseases. A total of 49 shared loci were mapped onto 11 protein-coding genes, and the main biological functions of the 11 shared genes were presented (Table 2). All of them were thought to modulate some critical physiological processes in human cells, including mitochondrial physiological mechanisms (e.g., *MTCH2*, *NDUFS3*, and *PTPMT1*), normal nuclear functions (e.g., *CELF1*, *MTCH2*, *NDUFS3*, *PSMC3*, and *PTPMT1*), an integral component of cellular membranes (e.g., *SPI1*, *FAM180B*, *MTCH2*, and *SLC39A13*), etc. These shared genes indicated a potential connection with the metabolic processes in human cells, and modulation of the expression of these genes might be related to the metabolic dysfunction and pathogenic issues. This result indicated the potential involvement of these shared genes in the development and progression of both AD and glaucoma.

**FIGURE 3:**
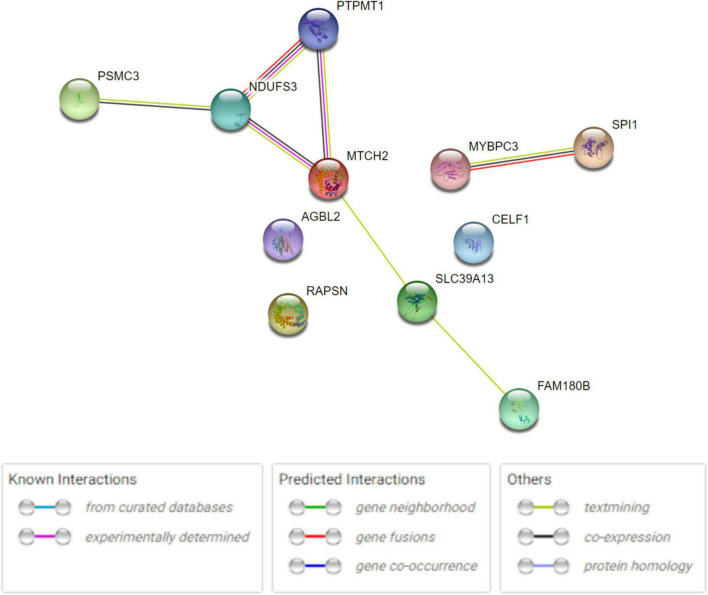
Human PPI network analysis for 11 shared genes between AD and glaucoma. PPI network connectivity for proteins identified in both AD and glaucoma. PPI results are based on the experimental evidence with the String database (https://string-db.org) (version 11.5). Network nodes represent proteins produced by the protein-coding gene loci. Edges between nodes indicate protein–protein interactions. The network contains 11 nodes with 7 edges.

The human PPI network analyses were conducted to investigate the potential networks in which the shared genes were enriched. The analysis revealed that these proteins have more interactions among themselves than expected for a random set of proteins of the same size and degree distribution drawn from the genome. This indicated that the shared proteins were at least partially biologically connected and carried out their functions concordantly (Figure 3), especially *MTCH2*, *NDUFS3*, and *PTPMT1*, as well as *SPI1* and *MYBPC3*. Metascape analysis revealed that *RAPSN*, *CELF1*, and *MTCH2* were considered to be enriched in the positive regulation of cell death pathway (GO:0010942, *P* < 0.05) (Supplementary Figure 2A). *NDUFS3*, *SPI1*, and *PTPMT1* were enriched in the apoptotic signaling pathway (GO:0097190, *P* < 0.05) (Supplementary Figure 2B). Analysis in DAVID functional annotation clustering showed that *MTCH2*, *NDUFS3*, and *PTPMT1* carried out their function on mitochondrion inner membrane (*P* < 0.01). COMPARTMENTS analysis indicated that the subcellular localization of most proteins was mapped on the cytosol (e.g., *AGBL2*, *MYBPC3*, *PSMC3*, and *RAPSN*), nucleus (e.g., *CELF1*, *MTCH2*, *NDUFS3*, *PSMC3*, *PTPMT1*, and *SPI1*), and mitochondria (e.g., *MTCH2*, *NDUFS3*, and *PTPMT1*).

### Expression of the pleiotropic genes shared by Alzheimer’s disease and glaucoma in human cells and tissues

**FIGURE 4:**
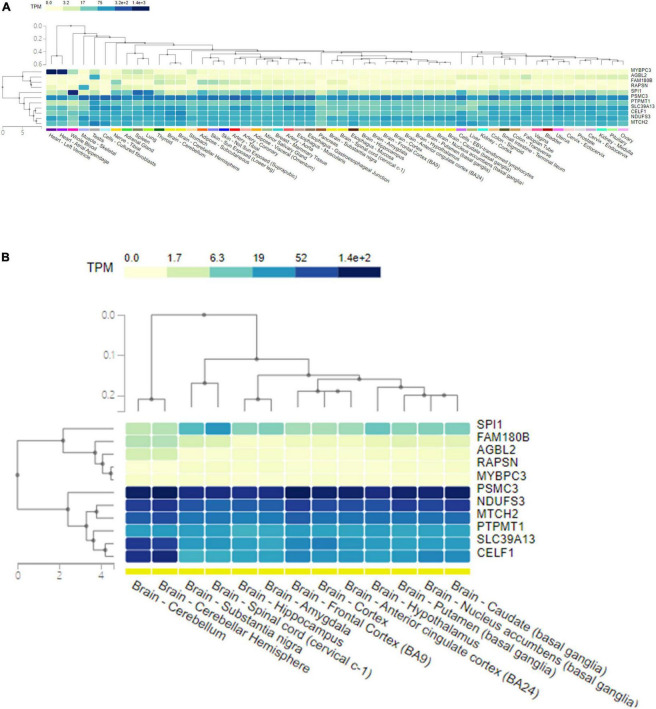
The expression level of 11 pleiotropic genes shared by AD and glaucoma in human tissues. **(A)** A heat map of the expression level of 11 pleiotropic genes shared by AD and glaucoma in all human tissues. The shared genes and the detected tissues were clustered by hierarchical clustering. The corresponding expression level of the gene in tissue was colored according to the linear count of the indicated TPM. **(B)** A heat map of the expression level of 11 pleiotropic genes shared by AD and glaucoma in human brain tissues. The shared genes and the detected brain tissues were clustered by hierarchical clustering. The corresponding expression level of the gene in brain tissue was colored according to the linear count of the indicated TPM. TPM represents transcript per million.

The GTEx database was used to present the expression levels of the shared genes in different human tissues, as well as their single-cell expression levels in various human cells and tissues. The genes and tissues detected were clustered by hierarchical clustering. The corresponding expression level was colored according to the linear count of the indicated transcript per million (TPM). Gene expression analysis of the shared pleiotropic genes in all human tissues showed that *MYBPC3* was mostly expressed in heart tissues, *SPI1* was mostly expressed in blood cells, and most of the shared genes (*CELF1*, *NDUFS3*, *PSMC3*, *PTPMT1*, *SLC39A13*, and *MTCH2*) were expressed homogenously across the human tissues (Figure 4A). When evaluating expression levels in human brain tissues individually, *CELF1*, *NDUFS3*, *PSMC3*, *PTPMT1*, *SLC39A13*, and *MTCH2* showed significantly higher expression levels than others (Figure 4B). *PSMC3* had a general higher expression level in all types of human brain tissues, while *CELF1* and *SLC39A13* showed higher expression in the cerebellum (Figure 4B). These results were in good agreement with the gene functions shown in Table 2.

**FIGURE 5:**
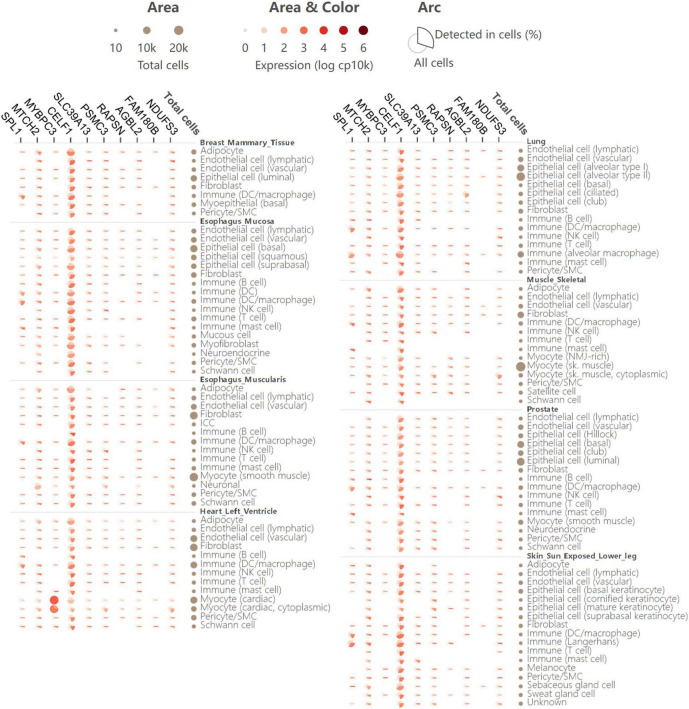
Single-cell expression analysis of 11 pleiotropic genes shared by AD and glaucoma in various human cell types and tissues. Single-cell expression levels of 11 shared pleiotropic genes in provided human cell types and tissues. In the matric format displayed as an aster plot, each row indicates one cell type from its tissue and each column indicates one gene. The arc area and color represent the comparison of the expression of all cells versus cells in which the gene is detected. The arc length presents the fraction of cells in which a gene is detected.

For further investigation of the expression atlas of pleiotropic genes in specific human cells and tissues, single-cell expression analysis was performed in the GTEx database. The single-cell transcriptomics provides an opportunity to identify significant roles of these genes in disease development and their cellular level heterogeneity. Comparison of gene expression in all human cells versus cells in which a gene was detected, as well as the fraction of cells in which a gene was detected, was also clearly illustrated in single-cell expression analysis (Figure 5). Generally, the 11 shared genes were expressed in all detected tissues. This means that in addition to being the risk loci of AD and glaucoma, the 11 shared genes might also take part in other diseases. *MYBPC3* was shown to have a higher expression in cardiac tissues and cells, which might indicate that *MYBPC3* modulates significant biological processes in myocytes. *CELF1* had rather homogenous expression in all detected human tissues and cells, which suggests that *CELF1* might participate in various common biological processes and owns important pathophysiological functions (Figure 5). As both *MYBPC3* and *CELF1* were found to play a role in other cells and diseases, they provide a good reference for further studies on AD and glaucoma. And there would be more similar mechanisms that also promote the development of these two neurodegenerative diseases.

## Discussion

Nowadays, aging and neurodegeneration are becoming increasingly prominent societal problems, putting great pressure on individuals and families alike (Yuzwa and Vocadlo, 2014; Mancino et al., 2018; Gupta et al., 2021; Nicholas et al., 2021). As typical representatives of neurodegenerative diseases, AD causes irreversible brain damage and glaucoma causes irreversible blindness (Quigley and Broman, 2006; Chang and Goldberg, 2012; Hansson, 2021). Recent research has demonstrated that there are connections between AD and glaucoma, with both being age-related neurodegenerative diseases that coexist in the aging population (Mirzaei et al., 2017; Giorgio et al., 2018; Mancino et al., 2018).

Currently, several clinical studies have revealed a significantly higher rate of glaucoma occurrence among AD patients (Chandra et al., 1986; Bayer et al., 2002a,b; Tamura et al., 2006; Helmer et al., 2013; Jefferis et al., 2013; Elyashiv et al., 2014; Mancino et al., 2018). In addition to numerous epidemiological studies, scientists have discovered pathophysiologic evidence to explain the potential mechanisms for the development and progression of the two neurodegenerative diseases. Among those pathological findings, Aβ and tau protein are commonly mentioned in many studies. They are thought to be closely related to metabolic dysfunction, ultimately causing chronic and progressive irreversible cell death (Lieven et al., 2007; Engelborghs et al., 2008; Sahara et al., 2008; Origlia et al., 2010; Gasparini et al., 2011; Poon et al., 2011; Bull et al., 2012; Della Santina et al., 2013; Gupta et al., 2014; Fá et al., 2016; Criscuolo et al., 2017). In AD, the aggregation and accumulation of Aβ and tau protein are then followed by a series of metabolic dysfunction processes, such as pro-inflammatory cytokines induction, reactive oxygen species production, oxidative stress development, and mitochondrial abnormalities (Origlia et al., 2010). Interestingly, in glaucoma, increased retinal levels of soluble Aβ protein also initiate the death of the retinal ganglion cells in the optic nerve head region, resulting in glaucoma (Poon et al., 2011; Della Santina et al., 2013; Gupta et al., 2014). Meanwhile, high intraocular pressure has also been proved to cause Aβ protein induction, leading to retinal degeneration (McKinnon et al., 2002; Guo et al., 2007). Another well-known characteristic of AD, tau protein, is also found in retinal ganglion cells, and increased phosphorylated tau protein production has been detected in aged retina and glaucoma (Lieven et al., 2007; Gasparini et al., 2011; Bull et al., 2012). Furthermore, the recent unifying “glymphatic system” hypothesis also integrates vascular, biomechanical, and biochemical theories to elucidate the potential existence of a paravascular transport system between the eye and the optic nerve in glaucoma. This theory has also been found in the human brain and is thought to play a role in AD (Jessen et al., 2015; Wostyn et al., 2015, 2017a,b). These findings suggest that AD and glaucoma should be studied together, and there may be potential inter-organ crosstalk between the brain and eye.

Given the limited understanding of AD, many drugs targeting Aβ and tau proteins have failed to provide clinical efficacy (Karran and De Strooper, 2022; Teipel et al., 2022). Other licensed symptom-relieved drugs, such as cholinergic pathway enhancers, glutamatergic transmission modulators, and disease-modifying drugs, still have limited clinical effects so far (Teipel et al., 2022). Currently, the only well-established management for glaucoma is lowering the intraocular pressure (Weinreb et al., 2014). However, despite successful medical and surgical treatments, severe complications, such as visual field loss, retinal ganglion cell death, and optic disc cupping, continue to worsen (Collaborative Normal-Tension Glaucoma, 1998; Jindal, 2013). Newly developed therapeutics, such as lipid nanoparticles, which are nanoparticulate systems, are indicated as a potential strategy for AD and glaucoma treatment (Sánchez-López et al., 2018). According to upcoming research, long-term nicotinamide treatment may also provide neuroprotective effect by protecting against mitochondrial and metabolic dysfunctions (Tribble et al., 2021).

Nevertheless, to treat or even prevent these two diseases, knowing their commonalities simply and superficially is not enough. This requires researchers to delve deeper into the basis of these diseases. Although the shared epidemiology and pathophysiology features of AD and glaucoma are usually mentioned, there are still limited understandings and insufficient explanations on the comorbidity, let alone the exploration of the shared mechanisms on genetic levels. With an increasing number of studies revealing the genetic factors affecting these two neurodegenerative diseases, investigating the genes and pleiotropies could help to further understand how genetic variants contribute to the development and association of the two diseases.

In this case, our study innovatively works to explore the associations between AD and glaucoma on genetic levels based on GWAS. We illustrated the genetic associations with a total of 49 genome-wide loci significantly shared between AD and glaucoma, which means that there were significant polygenetic overlaps between them. After mapping these loci onto 11 protein-coding genes, we explored their biological functions and investigated the potential mechanisms by which these genes modulate the diseases. Some important cellular processes, including those that occur in mitochondria, nucleus, and cellular membranes, were identified. And these shared genes have also been reported by other studies that mention their significant roles in neurodegenerative diseases. *SPI1* is involved in the genetic overlap between alcoholism and neurodegenerative diseases (Kapoor et al., 2021). It has also been reported to take part in the Aβ and tau pathology-induced astrocyte signature (Jiwaji et al., 2022). As a protein of the ubiquitin-proteasome degradation pathway, *PSMC3* has been shown to cause neurosensory syndrome by combing deafness and cataract (Kröll-Hermi et al., 2020). *NDUFS3* is reported to be associated with Aβ toxicity and is differentially expressed in the temporal cortex (Mukherjee et al., 2017). *MTCH2* is also involved in cardiovascular disease and AD (Broce et al., 2019).

In an attempt to explain the potential shared pathways of the two significant neurodegenerative diseases, human PPI network analyses were performed to determine whether there are certain pathways in which the shared genes are enriched. In STRING experimental results, our results found that some of the shared genes, particularly *MTCH2*, *NDUFS3*, and *PTPMT1*, as well as *SPI1* and *MYBPC3*, may function concordantly. However, when considering the pathway enrichment of these shared genes, no significant pathway enrichment was observed in the GO and KEGG pathways. This indicated that the shared pleiotropic genes were prone to carry out their function concordantly rather than functioning alone or enriched in certain pathways. By using Metascape pathway analysis, *RAPSN*, *CELF1*, and *MTCH2* were found to be enriched in the positive regulation of the cell death pathway (GO:0010942, *P* < 0.05). Similarly, *NDUFS3*, *SPI1*, and *PTPMT1* were found to be enriched in the apoptotic signaling pathway (GO:0097190, *P* < 0.05) (Supplementary Figure 2). However, the specific interactions between these proteins are not yet known. The COMPARTMENTS results indicated that proteins coded by some of these shared genes were enriched in certain subcellular locations of the cells. What is more, in gene expression analysis, some of these shared genes, such as *CELF1*, *NDUFS3*, *PSMC3*, *PTPMT1*, *SLC39A13*, and *MTCH2*, were shown to have higher expression levels than others in human brain tissues. All of the shared genes indicated a potential connection between the metabolic processes in human cells and tissues, such that the modulation of their expression might be related to metabolic dysfunction and pathogenic issues. The potential involvement of these shared genes in the development and progression of both diseases reflects the “inter-organ crosstalk” between AD and glaucoma.

In light of these findings, the comorbidity of AD and glaucoma require further studied. Our present study still has several limitations that require future investigation. For example, the data used in our study were mostly from European patients due to the limited number of published GWASs datasets, therefore the results from the general population and the potential racial discrepancies should be tested once they become available. Some epidemiology studies have suggested that older and female glaucoma patients were more likely to develop AD (Moon et al., 2018; Mullany et al., 2021), thus shared sex-specific genes are worth to be investigated since there would be some shared sex-specific effects at specific loci which are overshadowed by polygenic variance. However, owing to the limitations of the current GWAS datasets, age and sex subgroup analyses for shared genes could not yet be performed. Similarly, since there are some differences among different glaucoma subtypes on the epidemiology level, it would be better to perform genetic subgroup analyses among different subtypes of glaucoma. We would like to take this opportunity to sincerely call for a more complete GWAS dataset sharing for public use. What is more, the genes identified were not compared to other neurodegenerative diseases, and whether these genes are specific to AD and glaucoma comorbidity needs further research. Meanwhile, whether these genes are partially biologically connected or powerful enough to work individually requires future investigation. This suggests that the genetic factors underlying the mechanisms of diseases are more complex than currently believed. In this case, more datasets and experimental validation of individual risk loci are warranted in situations where the identification of causal variants is desired. Since AD and glaucoma have been linked in epidemiology, pathophysiology, and genomics, their clinical application needs to be prioritized. For example, glaucoma screening could be applied to AD patients to prevent future blindness. Similarly, cognitive status assessments could be performed annually to detect future AD development in glaucoma patients.

## Conclusion

In conclusion, we found encouraging evidence that AD and glaucoma share a genetic architecture that modulates their development and causes shared features in epidemiology and pathophysiology. Although these shared genes have not yet been well studied at the moment, they inspire us that there may be new mechanisms that have yet to be discovered, and that these genes themselves may play a significant role in the disease process individually. With a deeper cognition of the two diseases at the genetic and cellular levels, their shared genetic architecture is likely to become a popular area for research focused on developing novel and effective therapies for AD, glaucoma, and other neurodegenerative diseases.

## Data availability statement

The original contributions presented in the study are included in the article/Supplementary material, further inquiries can be directed to the corresponding authors.

## Author contributions

CZ, XLZ, and HY: study concept and design. CZ, XLZ, HY, MH, YJH, SL, XYZ, YYH, XS, ZZ, YH, GW, YX, ZD, YL, DC, and SZ: acquisition, analysis, or interpretation. CZ: drafting of the manuscript. XYZ, XLZ, SL, HY, and MH: critical revision of the manuscript for important intellectual content. CZ: manuscript revision. CZ, XLZ, and SL: statistical analysis. HY: obtained funding. XYZ, XLZ, XS, ZZ, YH, HY, and MH: administrative, technical, or material support. XLZ, HY, and MH: study supervision. All authors contributed to the article and approved the submitted version.

## Footnotes

1
https://www.ebi.ac.uk/gwas

2
https://github.com/KehaoWu/GWAScFDR

3
http://www.ncbi.nlm.nih.gov

4
https://string-db.org

5
https://metascape.org

6
https://www.gtexportal.org

## Supplementary material

Supplementary Figure 1 Workflow figure of the analysis processed of this study. Shared genetic architecture between AD and glaucoma was identified by performing pleiotropy analysis. The biological function and network analysis, and the expression in human tissues and cells of the shared genes identified were then analyzed. Figures were created with BioRender.com.

Supplementary Figure 2 Potential pathways of the shared genes between AD and glaucoma. **(A)** Positive regulation of cell death pathway (GO:0010942). **(B)** Apoptotic signaling pathway (GO:0097190).

Supplementary Table 1 Descriptive characteristics of GWAS summary datasets involved in this study.
