# Supplementary figures and images for: Shared genetic architecture between the two neurodegenerative diseases: Alzheimer’s disease and glaucoma
Source: Front Aging Neurosci. 2022 Sep 1;14:880576. doi: 10.3389/fnagi.2022.880576 (PMC9476600; doi:10.3389/fnagi.2022.880576)

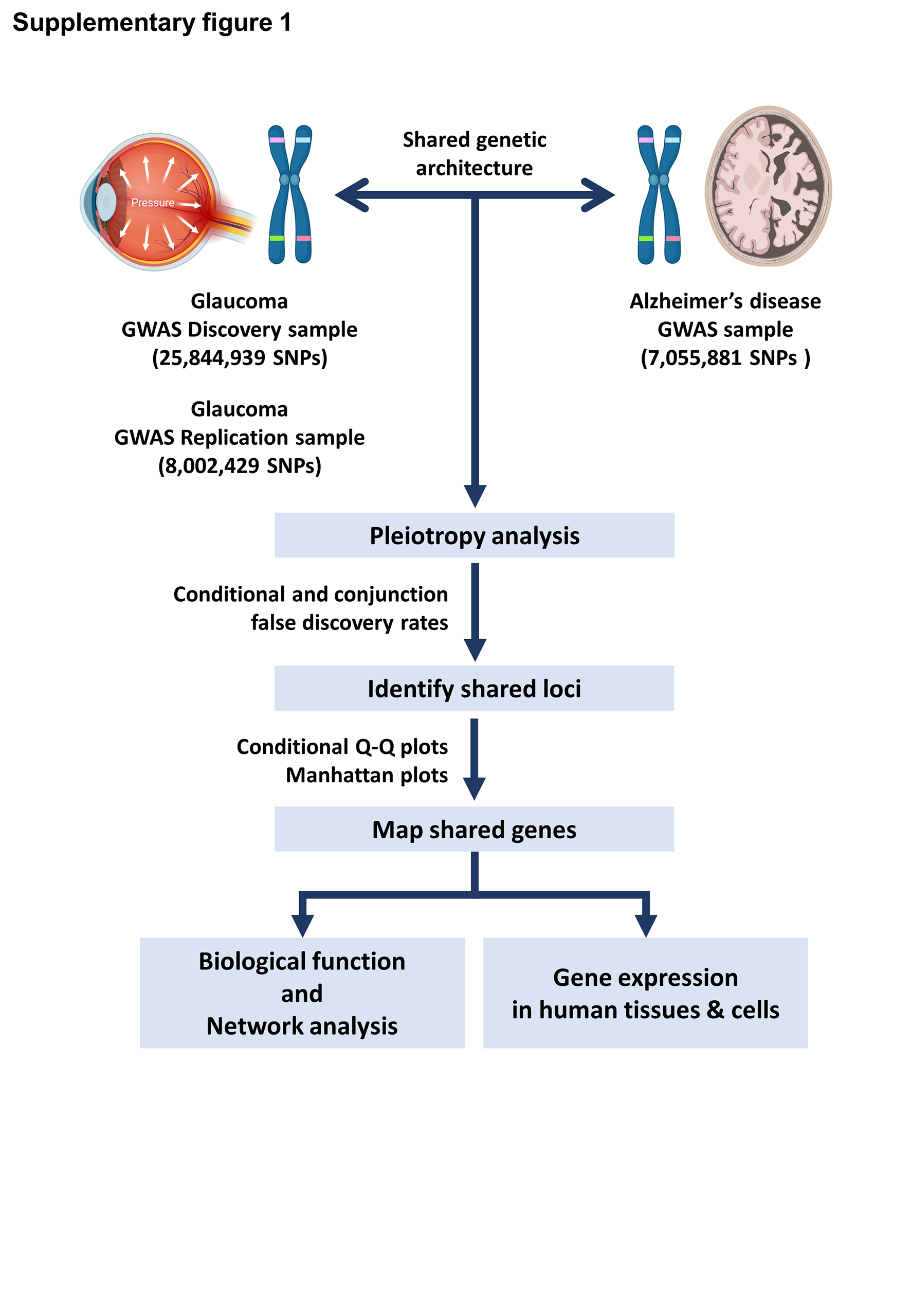

Supplement: Supplementary Figure 1 — Workflow figure of the analysis processed of this study. Shared genetic architecture between AD and glaucoma was identified by performing pleiotropy analysis. The biological function and network analysis, and the expression in human tissues and cells of the shared genes identified were then analyzed. Figures were created with BioRender.com. [file Image_1.TIF]

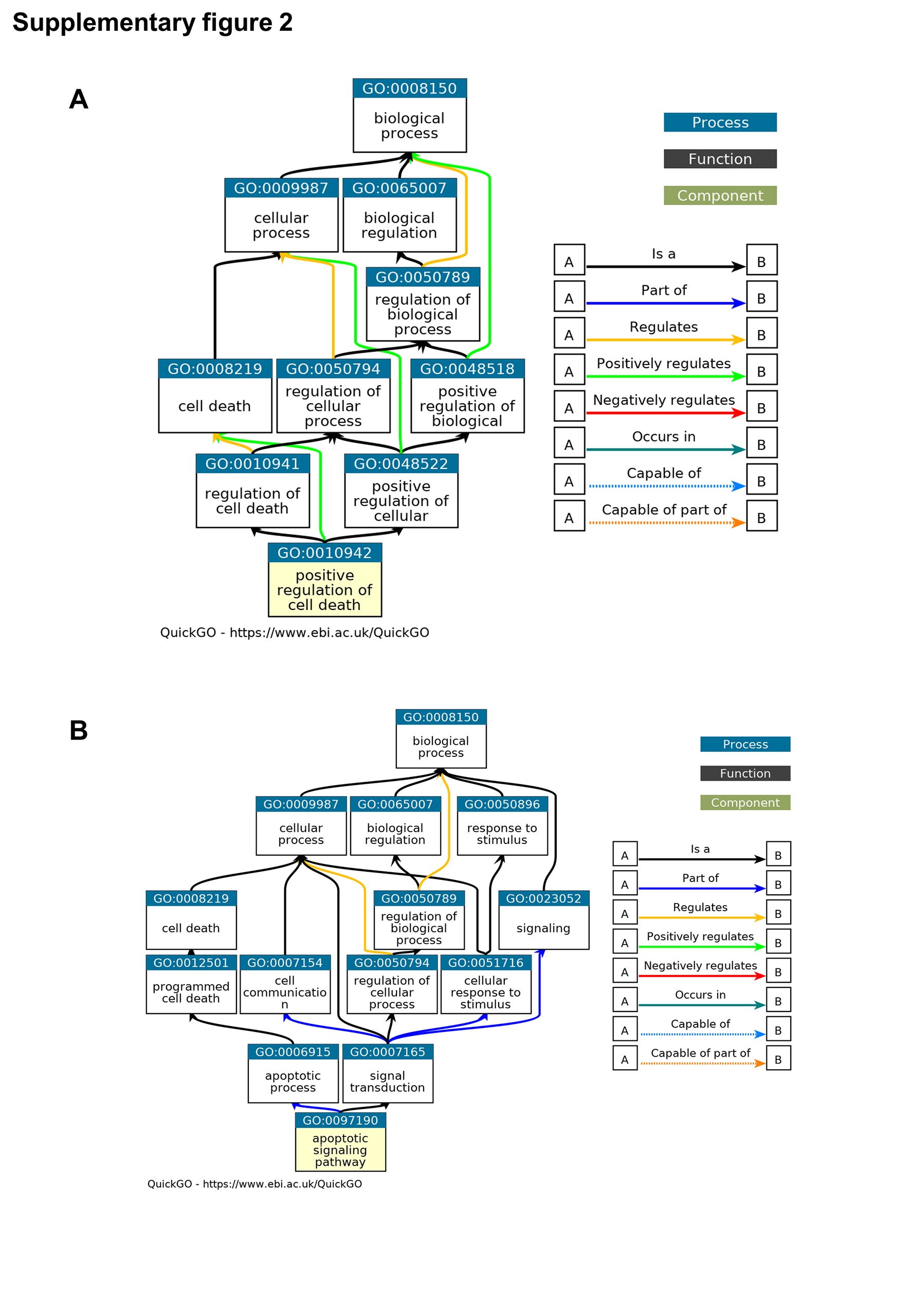

Supplement: Supplementary Figure 2 — Potential pathways of the shared genes between AD and glaucoma. (A) Positive regulation of cell death pathway (GO:0010942). (B) Apoptotic signaling pathway (GO:0097190). [file Image_2.TIF]
