# Supplementary material for: Shared genetic architecture between the two neurodegenerative diseases: Alzheimer’s disease and glaucoma
Source: Front Aging Neurosci. 2022 Sep 1;14:880576. doi: 10.3389/fnagi.2022.880576 (PMC9476600; doi:10.3389/fnagi.2022.880576)
Supplement: Supplementary Table 1 — Descriptive characteristics of GWAS summary datasets involved in this study. [file Table_1.DOCX]

**Supplementary table 1. Descriptive characteristics of GWAS summary datasets involved in this study.**

| **Traits** | **Sample Size**  **(cases/controls)** | **SNP numbers** | **Mean Age**  **(yrs)** | **Gender**  **(% Female)** | **References** |
| --- | --- | --- | --- | --- | --- |
| AD | 17,008/37,154 | 7,055,881 | 68.5-86.4 | 42.2%-75.0% | [49] |
| Glaucoma  (Discovery) | 18,859/643,471 | 25,844,939 | 56.8, 63.0 | 53.8%, 46.3% | [50] |
| Glaucoma  (Replication) | 7,947/119,318 | 8,002,429 | NA | NA | [51] |
